# Kinetics and thermodynamics evaluation of carbon dioxide enhanced oil shale pyrolysis

**DOI:** 10.1038/s41598-020-80205-4

**Published:** 2021-01-12

**Authors:** Shuai Zhao, Youhong Sun, Xiaoshu Lü, Qiang Li

**Affiliations:** 1 School of Mines, China University of Mining and Technology, Xuzhou, 221116 China; 2 School of Engineering and Technology, China University of Geosciences (Beijing), Beijing, 100083 China; 3 Construction Engineering College of JiLin University, Changchun, 130000 China; 4 Department of Electrical Engineering and Energy Technology, University of Vaasa, P.O.Box 700, N65101 Vaasa, Finland; 5 Department of Civil Engineering, Aalto University, P.O.Box. 11000, N02130 Espoo, Finland

**Keywords:** Geochemistry, Mineralogy

## Abstract

The pyrolysis process of oil shale is significantly affected by atmospheric conditions. In this paper, the pyrolysis experiments of oil shale under non-isothermal conditions are carried out using nitrogen and carbon dioxide as heat-carrying fluids. The results show that the activation energy of the second stage of oil shale pyrolysis under carbon dioxide is less than that under nitrogen. The thermodynamic analysis of the second stage of oil shale pyrolysis shows that Gibbs free energy, activation enthalpy and activation entropy are higher under carbon dioxide than those under nitrogen, which obeys the law of carbon dioxide promoting oil shale pyrolysis. In addition, the volatile release characteristics of oil shale in the second stage of pyrolysis were analyzed, which proves that the volatile release characteristics of oil shale under carbon dioxide are higher than that under nitrogen. Therefore, carbon dioxide is helpful to promote the pyrolysis of oil shale and increases the release of volatile substances during pyrolysis.

## Introduction

China is a big energy consumer. The long-term and rapid development of social economy cannot be separated from the energy consumption, but China relies too much on conventional energy, which leads to the dependence on foreign crude up to 69.8% in 2018 and endangers national energy security. Therefore, it is urgent to find alternative oil resources. Oil shale is an immature hydrocarbon-generating medium. It can produce oil and gas when the temperature reaches the pyrolysis temperature of kerogen^1^. The pyrolysis process of oil shale is a multi-phase and multi-stage coupled chemical reaction process^2, 3^. The pyrolysis of oil shale varies with areas, sedimentary characteristics and kerogen types. In addition, the choice of pyrolysis fluid medium will also play a key role. Studies have been conducted on the pyrolysis and combustion characteristics of oil shale and coal under various atmospheric conditions, such as nitrogen (N_2_) and oxygen-rich mixtures and stack gas. Bai et al. carried out the pyrolysis test of Huadian oil shale in oxygen-rich state. The results show that with the increase of oxygen concentration, the combustion performance of oil shale is significantly improved, in which the volatilization release temperature, ignition temperature and burnout temperature are reduced, the mass loss rate is increased, and the comprehensive combustion characteristics of oil shale are enhanced^4^. In order to explore the thermal efficiency and product composition of pulverized coal direct combustion, Li et al. carried out thermogravimetric tests of coal in the mixture of O_2_/CO_2_ and O_2_/N_2_. The experimental results show that the combustion process of pulverized coal in O_2_/CO_2_ environment is delayed compared in O_2_/N_2_ environment for the same oxygen concentration. Compared with the pulverized coal combustion in the environment of O_2_/N_2_, more CO (Carbon monoxide) is produced in the process of O_2_/CO_2_ combustion^5^. Zhou et al. studied the combustion characteristics of Yimin lignite and Jundong lignite in O_2_/CO_2_ by thermogravimetric analysis. The results show that the combustion performance can be significantly improved with the increase of oxygen concentration, especially when the oxygen concentration is less than 60%. For complex reaction process, the calculation of activation energy by isovolumetric transformation method can be used as a basis to judge the difficulty of reaction^6^. Lauri Loo et al. conducted a TG-DSC test on Estonian oil shale. Increasing oxygen ratio increases the overall combustion rate under oxygen fuel atmosphere. In addition, carbon dioxide (CO_2_) emissions are reduced by the reduction of carbonate decomposition^7^. Although many investigated CO_2_ in the process of the experiment, they focused on studying the role of oxygen concentration or the impact on CO_2_ emissions without studying deeply analyzing analyze the role of CO_2_ in the experiment.

CO_2_ can promote the transformation of semi-coke in coal and oil shale. For deep buried oil shale, CO_2_ can be used as heat-carrying fluid and displacement fluid to realize underground burial of CO_2_, which plays a positive role in reducing the greenhouse effect of the earth. Although scholars at home and abroad have done a lot of experiments on pyrolysis and combustion of lignite and oil shale under various atmospheric conditions, there are few studies on the effect of pure CO_2_ on the pyrolysis characteristics of oil shale. In this paper, Huadian oil shale is taken as a sample to study the pyrolysis kinetics, thermodynamic law and volatile release characteristic index of oil shale in pure CO_2_. It is verified that CO_2_ reduces the activation energy of oil shale pyrolysis and promotes the pyrolysis of oil shale.

## Material and principle

### Materials

The samples used in the experiment are from the fourth floor of Gonglangtou mining area in Huadian, Jilin Province, China. The results of Proximate, Fisher and elemental analysis are shown in Table 1.

**Table 1:** Analysis of oil shale in Huadian.

Attribute	Proximate analysis/wt%				Fisher analysis/wt%				Element analysis/wt%			
Region	Moisture	Ash	Volatiles	Fixed carbon	Shale oil	Water	Residue	Gas	H	C	N	S
HD1	4.15	49.71	44.37	1.77	19.16	3.45	66.32	11.07	4.271	29.96	0.64	2.094
HD2	4.12	50.26	44.24	1.38	18.31	3.79	67.39	10.51	4.02	28.86	0.64	2.12

### Thermogravimetry test method

**Figure 1:**
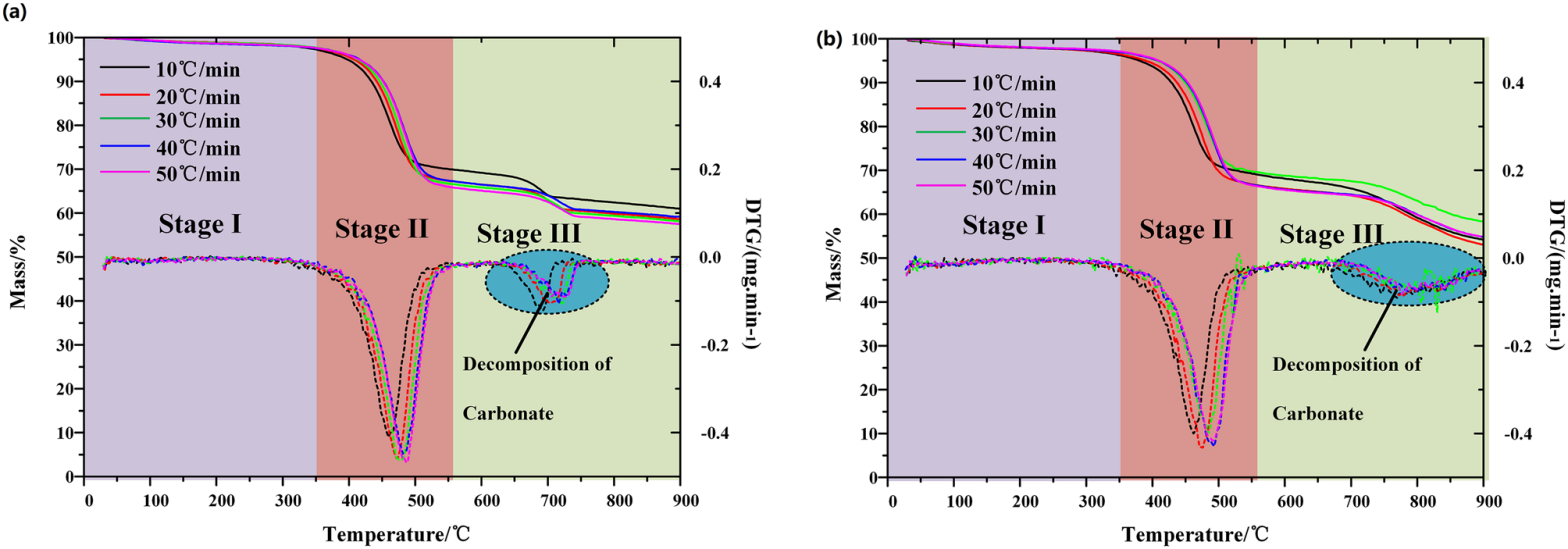
TG and DTG curve at (**a**) N_2_ and (**b**) CO_2_.

The weight of samples was 9.0 ± 0.1 mg, in which the initial temperature of TG curve was 25 °C with the heating rates of 10, 20, 30, 40, 50 °C/min and the final reaction temperature was 900 °C. The purge gas is N_2_ and CO_2_ with flow rate of 60 mL/min and the protective gas is in high purity N_2_ with flow rate of 25 mL/min. The TG-DTG curve under N_2_ and CO_2_ is shown in Fig. 1 below. In order to reproduce the experiment, each group of experiments was repeated at least twice.

As shown in Fig. 1, the first stage of weight loss is mainly the water loss. The water content of Huadian oil shale is low, and the free water content is lower than the bound water content. Therefore, the weight loss of oil shale samples is slow in this stage. In the second stage of weight loss, the pyrolysis rate of kerogen increased significantly, and the process of weight loss is rapid. With the increase of heating rate, the pyrolysis zone of kerogen moves to high temperature zone. Compared with N_2_ environment, the thermal hysteresis of oil shale pyrolysis under CO_2_ is more obvious. However, when the heating rate is higher than 40 °C/min, the thermal hysteresis decreases. The third weight loss stage is the decomposition stage of carbonate. The decomposition range of carbonate is more compact under N_2_. When carbonate decomposes, CO_2_ participates in the process, and promotes the decomposition of carbonate and widens the decomposition range of carbonate.

### Kinetic parameters calculations

#### KAS method for solving activation energy E_a_

KAS method is the abbreviation of Kissinger–Akahira–Sunose, and its calculation method is as follows^9^.

1 $$\mathrm{\alpha }=\frac{{m}_{0}-m}{{m}_{0}-{m}_{\infty }}$$

2 $$\mathrm{ln}\frac{\beta }{{T}^{2}}=\mathrm{ln}\left[\frac{A{E}_{a}}{G\left(a\right)R}\right]-\frac{{E}_{a}}{RT}$$

where, α—Conversion rate of oil shale pyrolysis in the second stage, %; m_0_—Initial mass of oil shale at the second pyrolysis stage, mg; m-sample mass at T K, mg; m_∞_—Final mass of oil shale at the second pyrolysis stage, mg; β—Heating rate, K min^−1^; T—reaction temperature, K; R—gas constant, 8.314 J mol^−1^; G(α)—Integral form of the most probable mechanism function; A—preexponential factor, s^−1^; E_a_—activation energy, kJ mol^−1^. It can be seen from Eq. (2) that $\mathrm{ln}\frac{\beta }{{T}^{2}}$ is a primary function of − $\frac{1}{T}$. By drawing $\mathrm{ln}\frac{\beta }{{T}^{2}}$ − (− $\frac{1}{T}$) at different conversion rates, the slope k can be obtained by fitting the curve with first order function. The activation energy E_a_ at the corresponding conversion rate can be obtained by k = E_a_/R.

#### FWO method for solving activation energy E_b_

In order to further verify the accuracy of KAS method, the Flynn–Wall–Ozawa method was used to calculate the apparent activation energy E_b_ again^10, 11^. The approximate formula of Doyle temperature integral was used, as shown in Eqs. (3) and (4).

3 $$\mathrm{P}\left(u\right)=0.00484 \cdot {e}^{-1.051u}$$

4 $$\mathrm{u}=\frac{{E}_{b}}{RT}$$

The temperature approximation is introduced into the pyrolysis integral Eq. (5).

5 $$\mathrm{G}\left(\alpha \right)=\frac{A{E}_{b}}{\beta R} \cdot P\left(u\right)$$

The calculation formula of FWO method can be obtained, as shown in Eq. (6).

6 $$\mathrm{lg\beta }=\mathrm{lg}\left(\frac{A{E}_{b}}{RG\left(\alpha \right)}\right)-2.315-0.4567\frac{{E}_{b}}{RT}$$

As can be seen from Eq. (6), lgβ is a first-order function of -$\frac{1}{T}$. Through drawing lgβ − (− $\frac{1}{T}$) at different conversion rates, the slope k can be obtained by fitting the curve with the first-order function. The activation energy E_b_ can be calculated by k = 0.4576E_b_/R.

#### Inference of the most probable mechanism function from y(α)-α

The values of data α_i_, y(α)_i_ (i = 0.05, 0.1……0.95) and α = 0.5, y(0.5) are brought into 41 groups of main functions of g(α) and corresponding f(α), as shown in Table 2.

**Table 2:** Kinetic mechanism function of solid-state reaction based on Malek method.

Number	Model	Differential form G(α)	Integral form f(α)
1	1D diffusion D_1_	α^2^	$\frac{1}{2}{\alpha }^{-1}$
2	2D diffusion D_2_	$\alpha +\left(1-\alpha \right)\mathrm{ln}\left(1-\alpha \right)$	${-\mathrm{ln}\left(1-\alpha \right)}^{-1}$
3,4	2D diffusion, n = 2, $\frac{1}{2}$	${\left[1-{\left(1-\alpha \right)}^{1/2}\right]}^{n}$	$m{\left(1-\alpha \right)}^{1/2}{\left[1-{\left(1-\alpha \right)}^{1/2}\right]}^{k}$ m = 4,1; k = $\frac{1}{2}$, − 1
5,6	3D diffusion n = 2, $\frac{1}{2}$	${\left[1-{\left(1-\alpha \right)}^{1/3}\right]}^{n}$	$\frac{3}{n}{\left(1-\alpha \right)}^{2/3}{\left[1-{\left(1-\alpha \right)}^{1/3}\right]}^{1-n}$
7	2D diffusion, n = 2, $\frac{1}{2}$	$\left(1-\frac{2}{3}\alpha \right)-{\left(1-\alpha \right)}^{2/3}$	$\frac{3}{2}{\left[{\left(1-\alpha \right)}^{-1/3}-1\right]}^{-1}$
8	3D diffusion	${\left[{\left(1+\alpha \right)}^{1/3}-1\right]}^{2}$	$\frac{3}{2}{\left(1+\alpha \right)}^{2/3}{\left[{\left(1+\alpha \right)}^{1/3}-1\right]}^{-1}$
9	3D diffusion	${\left[{(1/\left(1+\alpha \right)}^{1/3})-1\right]}^{2}$	$\frac{3}{2}{\left(1-\alpha \right)}^{4/3}{\left[{\left(1-\alpha \right)}^{-1/3}-1\right]}^{-1}$
10 ~ 15, 17 ~ 20	Avrami-Erofeev n = $\frac{2}{3}$,$\frac{1}{3}$,$\frac{1}{2}$,$\frac{1}{4}$,4,$\frac{3}{4}$,$\frac{3}{2}$,2,3,$\frac{2}{5}$	${\left[1-ln\left(1-\alpha \right)\right]}^\frac{1}{n}$	$n\left(1-\alpha \right)\times [{-ln\left(1-\alpha \right)]}^{1-\frac{1}{n}}$
16	Mample rule	$-\mathrm{ln}\left(1-\alpha \right)$	$1-\alpha$
21	Autocatalytic reaction	$\mathrm{ln}\left(\frac{\alpha }{1-\alpha }\right)$	$\alpha \left(1-\alpha \right)$
22 ~ 24, 26 ~ 27	Mample Power rule n = 2,$\frac{1}{3}$,$\frac{1}{2}$,$\frac{1}{4}$,$\frac{3}{2}$	${\alpha }^{n}$	$\frac{1}{n}{\left(\alpha \right)}^{1-n}$
25, 28, 29, 31,33 ~ 35	Phase boundary reaction n = $\frac{1}{3}$,$\frac{1}{2}$,$\frac{1}{4}$,4,2,3,1	$1-{\left(1-\alpha \right)}^{n}$	$\frac{1}{n}{\left(1-\alpha \right)}^{1-n}$
30	3D Phase boundary reaction	$3\left[1-{\left(1-\alpha \right)}^{1/3}\right]$	${\left(1-\alpha \right)}^{2/3}$
32	2D Phase boundary reaction	$2\left[1-{\left(1-\alpha \right)}^{1/2}\right]$	${\left(1-\alpha \right)}^{1/2}$
36	Reaction order, F_2_, α-t Deceleration form	${\left(1-\alpha \right)}^{-1}$	${\left(1-\alpha \right)}^{2}$
37	Reaction order, F_2_,	${\left(1-\alpha \right)}^{-1}-1$	${\left(1-\alpha \right)}^{2}$
38	Reaction order, F_2/3_	$(1-\alpha {)}^{-1/2}$	${2\left(1-\alpha \right)}^{3/2}$
39	α-t Accelerated form; E_1_	$\mathrm{ln}\alpha$	α
40	Exponential rule	$\mathrm{ln}{\alpha }^{2}$	$\frac{1}{2}\alpha$
41	Reaction order, F_3_,	${\left(1-\alpha \right)}^{-2}$	$\frac{1}{2}{\left(1-\alpha \right)}^{3}$

The main function was calculated by y(α)^12^, and the curve of y(α)-α is plotted, which is regarded as the standard curve.

7 $$\mathrm{y}\left(\mathrm{\alpha }\right)=\frac{f(\alpha ) \cdot G(\alpha )}{f(0.5) \cdot G(0.5)}$$

Then, the experimental data α_i_, T_i_, ${\left(\frac{d\alpha }{dt}\right)}_{i}$(i = 0.05, 0.1……0.95) and α = 0.5, T_0.5_,${\left(\frac{d\alpha }{dt}\right)}_{0.5}$ were inserted into Eqs. (7) and (8) was obtained. The corresponding calculation of y(α) was also carried out, and the test curve of y(α)-α was drawn on the basis of the standard curve.

8 $$\mathrm{y}\left(\mathrm{\alpha }\right)={\left(\frac{T}{{T}_{0.5}}\right)}^{2} \cdot \frac{\left(\frac{d\alpha }{dt}\right)}{{\left(\frac{d\alpha }{dt}\right)}_{0.5}}$$

If the test curve overlaps with the standard curve, or the data points of the test curve all fall on a certain standard curve. It can be determined that f(α) and g(α) corresponding to the curve are the most probable kinetic mechanism functions.

**Figure 2:**
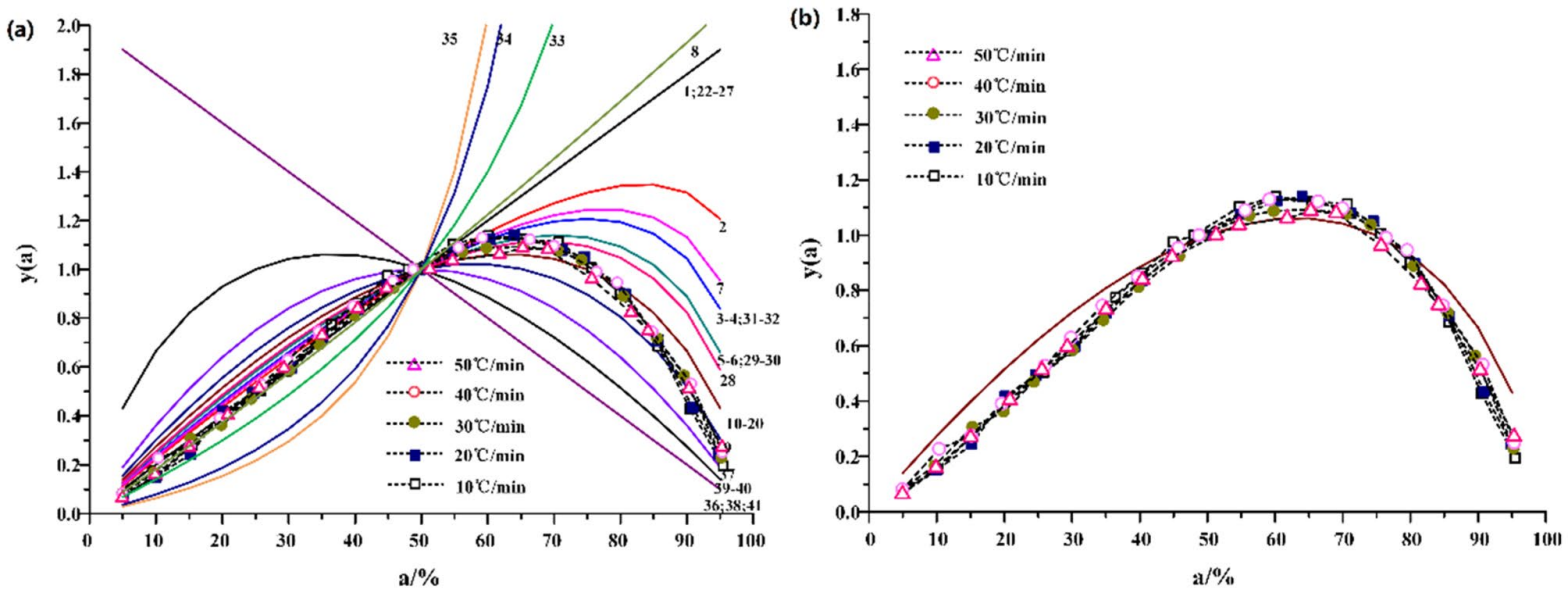
Standard and experimental curves of y(α)-α under N_2_.

**Figure 3:**
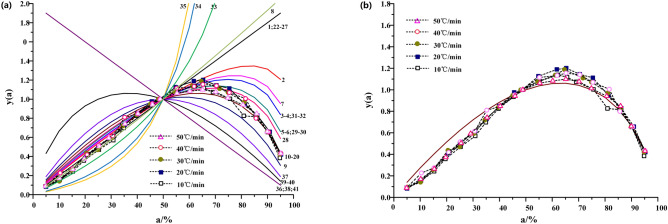
Standard and experimental curves of y(α)-α under CO_2_.

The standard curves and experimental curves of 41 candidate mechanism functions y(α)-α are plotted by melak method as shown in Figs. 2 and 3. The pyrolysis curves of Huadian oil shale under N_2_ and CO_2_ are distributed near Johnson-Mehl-Averami equation when the heating rate is between 10 °C/min and 50 °C/min. It shows that the pyrolysis mechanism function of Huadian oil shale satisfies the reaction mechanism of random growth and subsequent nucleation. However, for the Johnson–Mehl–Averami reaction model, there are many different kinetic indices n. The pre-exponential factor A and other thermodynamic parameters can be estimated only if the kinetic exponent n is known exactly. For Johnson–Mehl–Averami reaction mode^13^,

9 $$\mathrm{G}\left(\mathrm{\alpha }\right)={\left[1-ln\left(1-\alpha \right)\right]}^\frac{1}{n}$$

10 $$f\left(\mathrm{\alpha }\right)=\mathrm{n}\left(1-\mathrm{\alpha }\right)\times [{-ln\left(1-\alpha \right)]}^{1-\frac{1}{n}}$$

Combining with DTG curve, when the reaction rate reaches the maximum value,

11 $$-{f}^{^{\prime}}\left({\alpha }_{P}\right) \cdot \mathrm{G}\left({\alpha }_{P}\right)=1$$

12 $${f}^{^{\prime}}\left({\alpha }_{P}\right)=\left(n-1\right)[{-ln\left(1-{\alpha }_{P}\right)]}^{-\frac{1}{n}}-n{\left[-ln\left(1-{\alpha }_{P}\right)\right]}^{1-\frac{1}{n}}$$

where, ${\alpha }_{P}$ is the corresponding conversion at the peak of DTG curve, %. Therefore, the kinetic exponent n can be obtained by combining the above Eqs. (9)–(11).

13 $$\mathrm{n}=\frac{1-{u}_{P}\pi ({u}_{P})}{ln\left(1-{\alpha }_{P}\right)+1}$$

14 $${u}_{P}=\frac{{E}_{P}}{R{T}_{P}}$$

where, ${E}_{P}$ is the activation energy corresponding to the peak value of DTG curve, J mol^−1^; ${T}_{P}$ is the temperature corresponding to the peak value of DTG curve, K. According to Luke approximation^13^,

15 $$\uppi \left({u}_{P}\right)=\frac{{u}_{P}^{3}+{18u}_{P}^{2}+86{u}_{P}+96}{{u}_{P}^{4}+{20u}_{P}^{3}+{120u}_{P}^{2}+{240u}_{P}+120}$$

### Solution of thermodynamic parameters

Because the activation energy calculated by KAS method is similar to that calculated by FWO method, the data calculated by either method can be used to evaluate the thermodynamic parameters. In this paper, the activation energy data calculated by KAS method is selected. Activation energy E and pre-exponential factor A obtained by kinetic calculation are substituted into Eq. (16) respectively. The k is Arrhenius constant, and it is the slope of the KAS plot. So, Arrhenius constant k^14^ at different heating rates can be obtained.

16 $$\mathrm{lnk}=\mathrm{lnA}-\frac{E}{RT}$$

Then, the Arrhenius constant k is introduced into Eq. (17) to obtain ΔG^≠^ at different heating rates.

17 $$\Delta {G}^{\ne }=RTln\frac{RT}{Nhk}$$

where, T is the experimental temperature, K; R is the gas molar constant, 8.314 J mol^−1^ K^−1^; N is the Avogadro constant, 6.024 × 10^23^ mol^−1^; h is the Planck constant, 6.625 × 10^−35^ J s.

From the Eyring equation,

18 $$\mathrm{ln}\frac{k}{T}=\left(\frac{{\Delta S}^{\ne }}{R}+ln\frac{{k}_{B}}{h}\right)-\frac{{\Delta H}^{\ne }}{RT}$$

where, k_B_ is a Boltzmann constant, 1.3807 × 10^–23^ J K^−1^. The curve of $\mathrm{ln}\frac{k}{T}$ − $\frac{1}{T}$ can be plotted. The ${\Delta H}^{\ne }$ can be obtained from the slope of the straight line. From the intercept of the straight line ${\Delta S}^{\ne }$ can be obtained.

## Results and discussion

### Apparent activation energy

**Figure 4:**
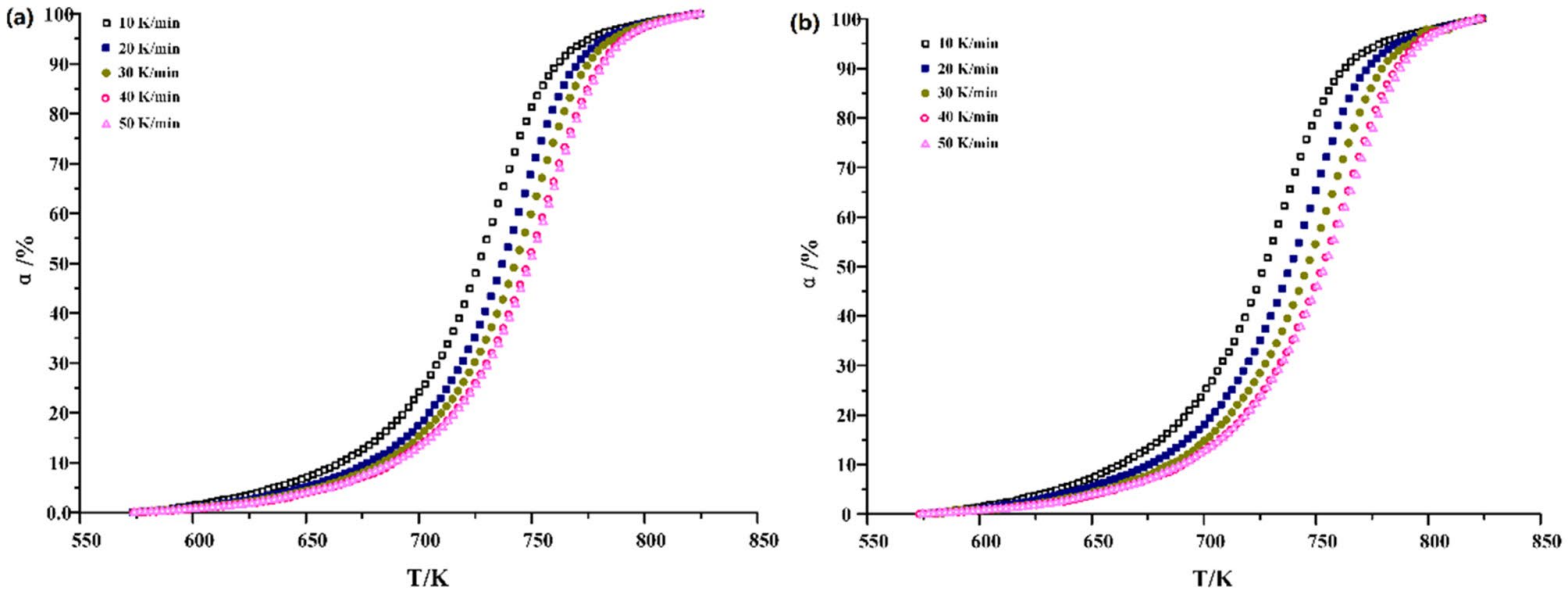
Conversion of oil shale pyrolysis in the second stage under (**a**) N_2_ and (**b**) CO_2_.

The second stage of oil shale pyrolysis is regarded as "oil window". The activation energy of oil shale in this stage will directly affect the difficulty degree of pyrolysis reaction^15^. As shown in Fig. 4, the conversion rate varies with temperature and heating rate under different atmospheric conditions.

**Figure 5:**
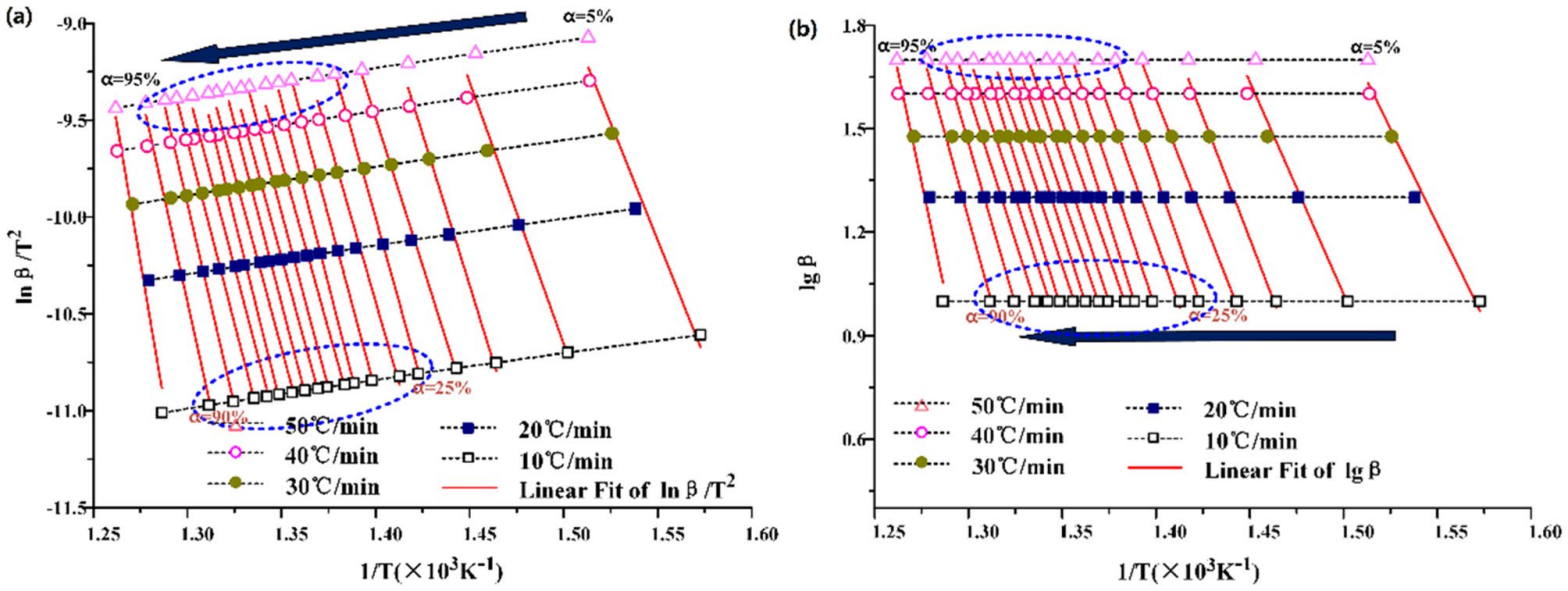
Calculating curve of activation energy under N_2_.

**Figure 6:**
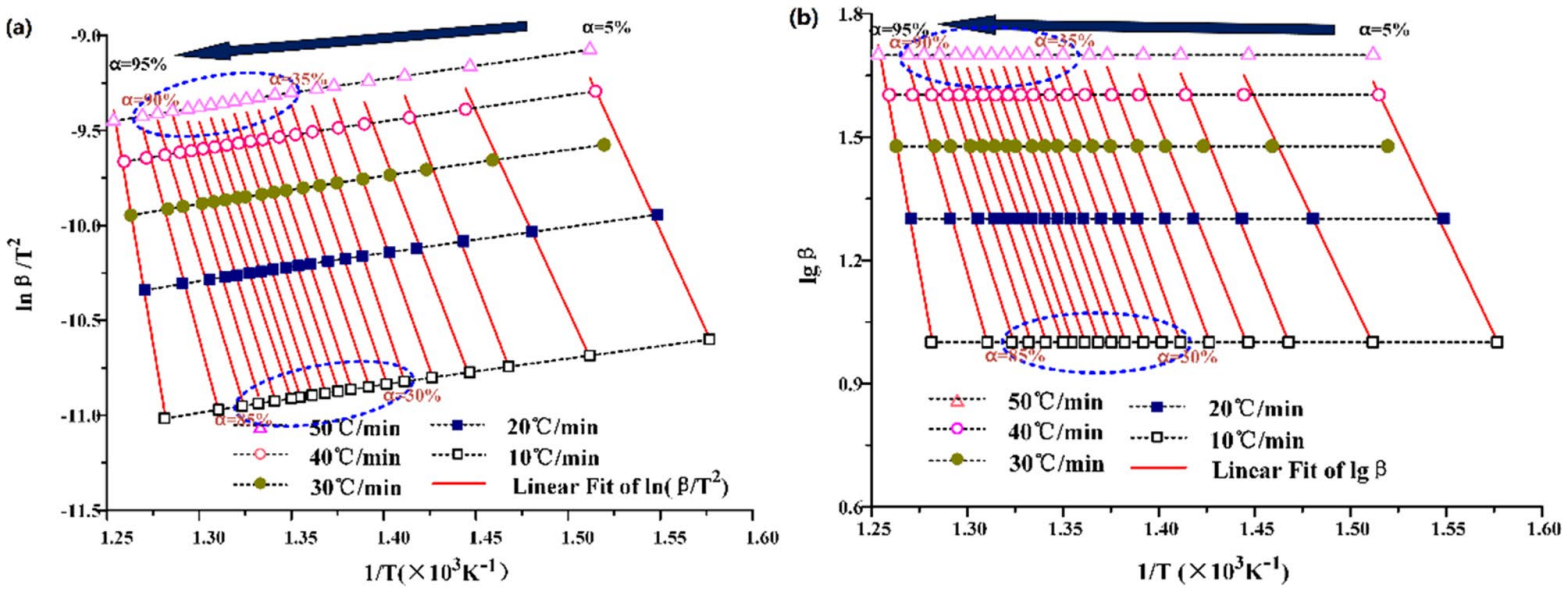
Calculating curve of activation energy under CO_2_.

KAS and FWO methods are used to calculate the kinetic parameters of the second stage of Huadian oil shale pyrolysis under the two atmospheric conditions. As shown in Figs. 5 and 6 below, the correlation coefficients r calculated by KAS and FWO methods are greater than 0.98, and the data fluctuation is small during the fitting process. This shows that KAS and FWO methods are suitable for the calculation of the pyrolysis kinetics of Huadian oil shale.

The process curves of activation energy calculated by the two methods show that under N_2_, the conversion rate of oil shale pyrolysis concentration ranges from 25 to 90%, and the corresponding temperature ranges from 700 to 774 K when the heating rate is low. The maximum weight loss rate is achieved at the conversion rate 48%. With the increase of heating rate, the conversion rate of oil shale pyrolysis becomes 35% ~ 90%. Under CO_2_, the conversion of oil shale pyrolysis is between 30 and 85%, and the corresponding temperature is between 706 and 745 K when the heating rate is low. The maximum weight loss rate is achieved at the conversion rate 45%. With the increase of heating rate, the conversion rate of oil shale pyrolysis is 35% ~ 90%. The reaction rate equation showed

19 $$\frac{d\alpha }{dt}=\frac{d\alpha }{dT}\frac{dT}{dt}=\beta \frac{d\alpha }{dT}=kg\left(\alpha \right)$$

20 $$\frac{d\alpha }{dT}=\frac{kg\left(\alpha \right)}{\beta }$$

This equation showed that the differential (or the rate) based on the temperature is lower when the heating rate is higher. Therefore, the conversion rate should be lower when the heating rate is higher. Oil shale belongs to heterogeneous system. In the process of pyrolysis, there is an active region of reaction area at the interface between reactant and product. The energy provided by the system for this region is larger than the activation energy of oil shale pyrolysis, so the oil shale in the active region is pyrolyzed first^16^. With the increase of heating rate, the scale of temperature rise per unit time increases. Increased temperature will first be reflected in the reactive region. The oil shale in the active region undergoes random growth of products and subsequent nucleation of interfacial reactions. Although the temperature rises rapidly, the interfacial reaction in the active region is not completed quickly, and the continuous progress of pyrolysis process takes time. Therefore, the interfacial reaction in the active region has to be promoted to a high conversion rate gradually with the increase of the heating rate.

Based on Eqs. (9)–(15), the calculated kinetic index n of oil shale pyrolysis is shown in Table 3. Wei Wang et al. used thermogravimetric analysis to study the thermodynamic behavior of North Korean oil shale under non-isothermal conditions^8^. It was found that the kinetic reaction order of North Korean oil shale was 1.5.

**Table 3:** Calculations of dynamics index.

β/°C min^−1^	10/°C min^−1^	20/°C min^−1^	30/°C min^−1^	40/°C min^−1^	50/°C min^−1^	Average
CO_2_	1.477	1.425	1.432	1.503	1.504	1.468
N_2_	1.520	1.513	1.402	1.513	1.569	1.503

By substituting the kinetic exponent n into the kinetic calculation Eqs. (2) and (6) using KAS method and FWO method respectively, the pre-exponential factor A and activation energy E can be calculated. As shown in Tables 4 and 5.

**Table 4:** Preexponential factors and activation energies under N_2_.

α/%	10	20	30	40	50	60	70	80	90
D	29.83	33.23	36.55	41.64	38.91	39.78	41.68	39.23	46.35
G (a)	1.07	1.14	1.22	1.32	1.42	1.54	1.69	1.89	2.21
r	0.946	0.997	0.9801	0.996	0.992	0.995	0.985	0.999	0.969
E/(kJ mol^−1^)	224.39	253.68	279.02	314.37	301.04	309.35	324.36	312.30	363.17
A	0.36E+08	1.02E+10	2.75E+11	4.28E+13	3.12E+12	7.96E+12	5.5E+13	5.5E+12	6.91E+15

**Table 5:** Preexponential factors and activation energies under CO_2_.

α/%	10	20	30	40	50	60	70	80	90
D	21.06	25.82	30.16	29.12	29.57	29.66	28.63	31.46	36.47
G(a)	0.22	0.36	0.49	0.63	0.78	0.94	1.13	1.38	1.76
r	0.968	0.968	0.971	0.991	0.991	0.990	0.991	0.992	0.988
E/(kJ mol^−1^)	174.39	210.46	241.45	238.67	244.47	247.65	243.58	264.37	301.00
A	14,427.88	0.23E+08	2.15E+08	0.97E+08	1.85E+08	2.4E+08	1.05E+08	2.01E+09	3.36E+11

**Figure 7:**
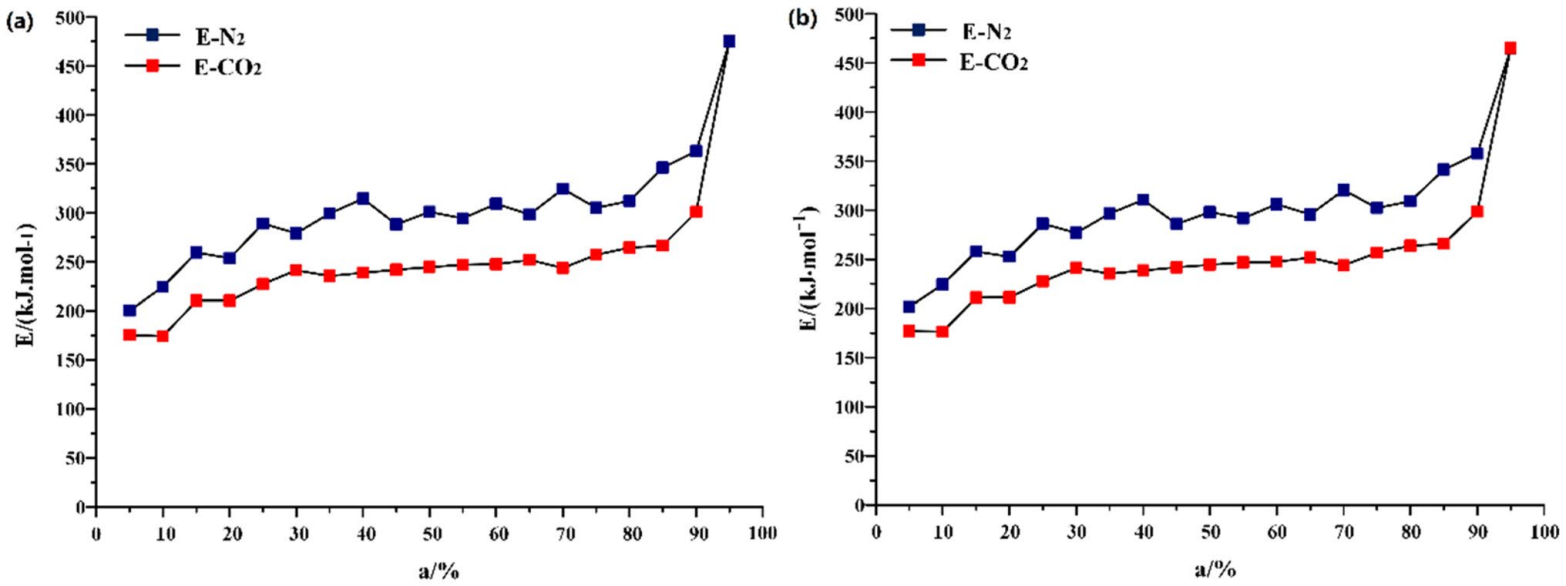
Activation energy of Huadian oil shale pyrolysis during the stage II (**a**) KAS (**b**) FWO.

The activation energy in the main oil production stage is of great significance to the whole process and industrial production of oil shale pyrolysis. As shown in Fig. 7, the activation energies calculated by KAS and FWO methods show an increasing trend with the increase of conversion, and the values of the two methods are almost the same.

It is noteworthy that in the pyrolysis stage with conversion of 20% to 90%, the activation energies under the two atmospheric conditions will have a relatively stable value. Under N_2_, the activation energy is about 300 kJ mol^−1^, but under CO_2_, the activation energy is only about 240 kJ mol^−1^. When the conversion reaches 90%, the activation energy increases sharply. At last, the activation energy will reach 460 kJ mol^−1^ in both N_2_ and CO_2_. However, in the actual industrial production, the conversion rate is difficult to achieve more than 90%, so CO_2_ can reduce the activation energy of oil shale in the oil-generating stage and promote the pyrolysis of oil shale is accurate. The activation energies obtained by KAS and FWO methods are relatively stable and have the same trend. The average activation energy of the second stage of oil shale pyrolysis under N_2_ is about 301.96 kJ mol^−1^, and that under CO_2_ is about 250.28 kJ mol^−1^.

According to the transition state theory, the pyrolysis of oil shale is controlled by the mechanism of interfacial reaction. At different heating rates, even a very little samples will have heat and mass transfer delay, which is caused by the transmission resistance formed by the accumulation of oil shale particles. The transport resistance also leads to the incomplete pyrolysis of oil shale and formats semi coke. The more cokes on the oil shale surface adhere, the more likely to lead to the increase of activation energy. Jacques LéDé has the same conclusion^17^. Under nitrogen atmosphere, oil shale is mainly pyrolyzed, while the accumulation of oil shale particles and the uneven heat and mass transfer easily lead to a large number of semi cokes. Moreover, the specific heat capacity and thermal conductivity of nitrogen are higher than that of carbon dioxide, which means that increasing the same temperature will consume more heat. Therefore, in TG test, it is not conducive to the heat conduction of oil shale. This is also a reason for the increase of activation energy.

### Thermodynamic decomposition characteristics

According to the transition state theory, kerogen needs to undergo a transition state in the pyrolysis process of oil shale to produce oil and gas. During this transition, energy is redistributed and chemical bonds are rearranged. By calculating the changes of activation enthalpy ΔH^≠^ activation entropy ΔS^≠^ and Gibbs free energy ΔG^≠^, the difficulty, spontaneity and reaction heat of thermal decomposition of oil shale can be understood. It can provide reference for industrialized in-situ pyrolysis of oil shale.

**Figure 8:**
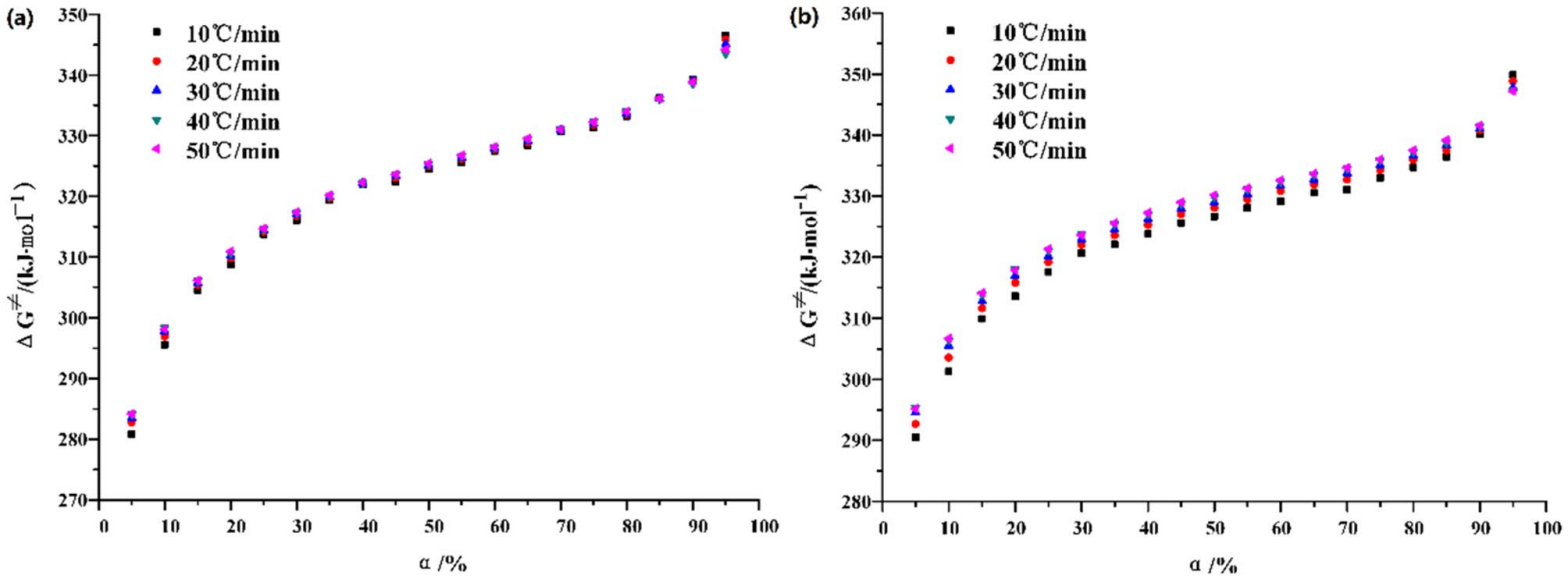
Transitional Gibbs free energy ΔG^≠^ varies with conversion (**a**) N_2_ and (**b**) CO_2_.

The calculation results of Eq. (17), it is pointed out that the Gibbs free energy ΔG^≠^ varies with the heating rate and conversion rate. As shown in Fig. 8, with the increase of heating rate, ΔG^≠^ shows an upward trend, but this upward scale is weak. With the increase of conversion and temperature, the ΔG^≠^ increases, which is consistent under N_2_ and CO_2_. Under N_2_, the corresponding conversion rate of oil shale pyrolysis concentrated in different stages ranged from 25 to 90%, and the ΔG^≠^ between these zones is estimated to be 326.86 kJ mol^−1^. However, under the atmosphere of CO_2_, the corresponding conversion rate of oil shale pyrolysis concentrated stage is 30% ~ 90%. The ΔG^≠^ between these zones is calculated to be 331.26 kJ mol^−1^.

**Figure 9:**
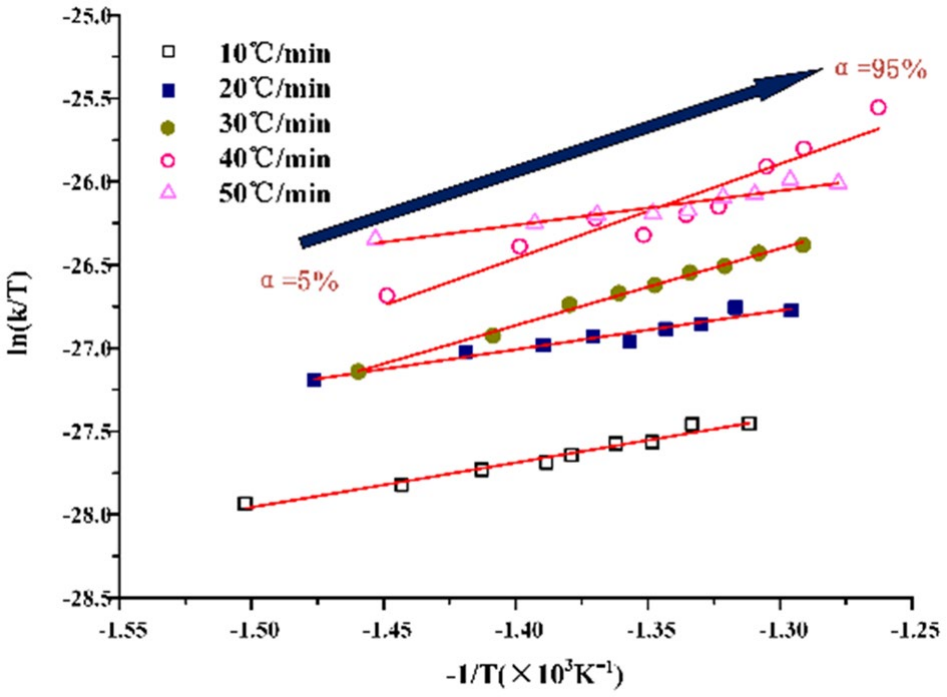
Calculating curves of activation enthalpy ΔH^≠^ and activation entropy ΔS^≠^ under N_2_.

**Figure 10:**
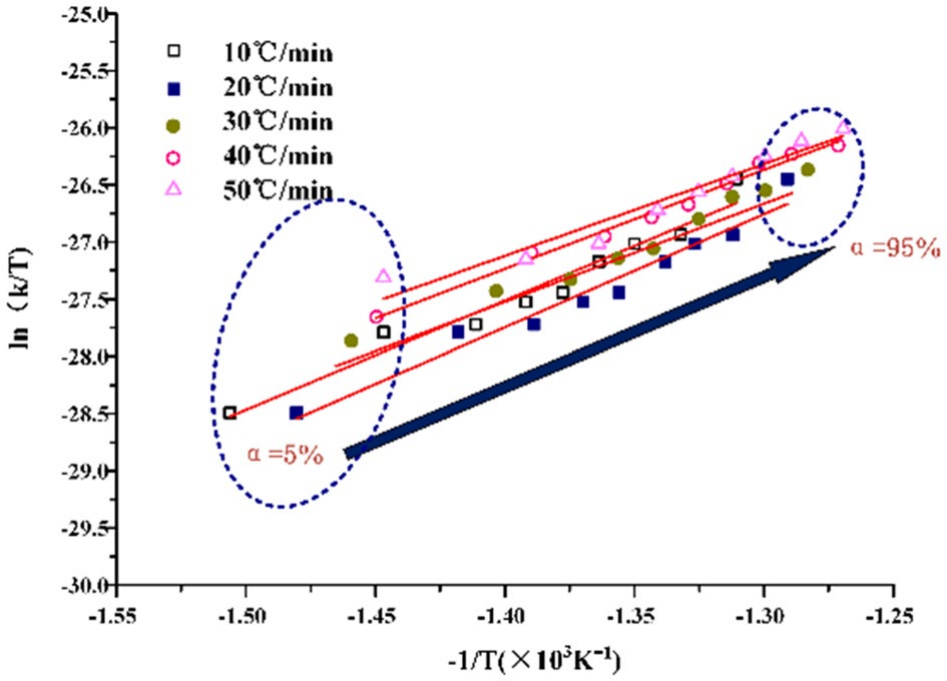
Calculating curves of activation enthalpy ΔH^≠^ and activation entropy ΔS^≠^ under CO_2_.

In the process of calculating activation enthalpy ΔH^≠^ and activation entropy ΔS^≠^ by mapping $\mathrm{ln}\frac{k}{T}$ − $\frac{1}{T}$, it is found that the data fitting curve has strong regularity, as shown in Figs. 9 and 10. The experimental data can be distributed on the fitting curve, and the fluctuation is small. Under N_2_, the fitting data are greatly affected by the heating rate, and the discretization degree between the heating rates is greater. Under CO_2_, it can be seen that the degree of data aggregation is higher, but the degree of discretization is higher at the stage of low conversion rate, indicating that oil shale pyrolysis is more stable under CO_2_ although the heating rate will also have an impact.

**Figure 11:**
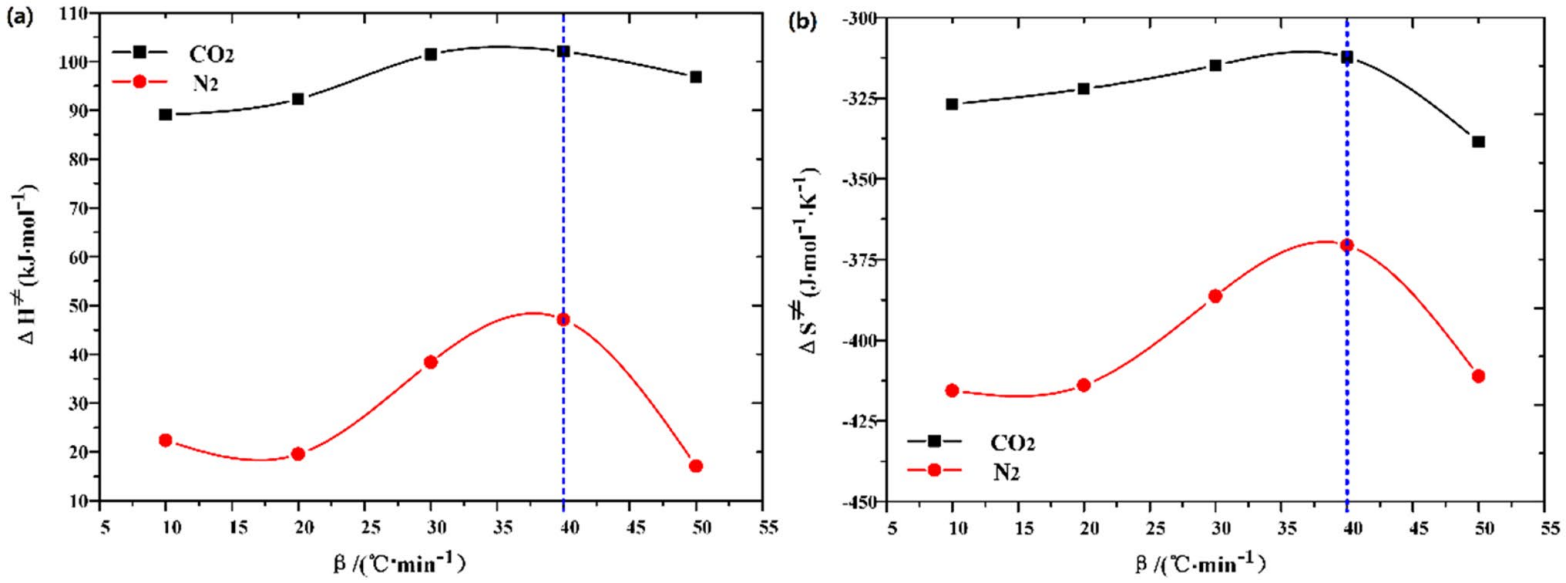
(**a**) Activation enthalpy and (**b**) activation entropy varies with heating rate.

Based on Eq. (18) and ${\text{ln}}\frac{k}{T} - \frac{1}{T}$ curve, the activation enthalpy ΔH^≠^ and activation entropy ΔS^≠^ are calculated, as shown in Fig. 11.

The variation of activation enthalpy with heating rate is shown in Fig. 11a. The second stage of Huadian oil shale pyrolysis has endothermic process to varying degrees under the atmospheric conditions of both N_2_ and CO_2_ pyrolysis. And with the increase of heating rate, the heat absorption increases. However, when the heating rate is higher than 40 ℃/min, the heat absorption decreases, which also proves that when the heating rate is higher than 40 °C/min, the influence of heating rate on the pyrolysis process of oil shale is weakened, that is, the thermal hysteresis phenomenon is weakened. The average activation enthalpy under N_2_ is about 28.98 kJ mol^−1^, and about 76.35 kJ mol^−1^ under CO_2_.

In addition, as shown in Fig. 11b, the activation enthalpy ΔH^≠^ and activation entropy ΔS^≠^ under both atmospheric conditions increase with the increase of heating rate, indicating that the process can proceed spontaneously at high temperature. However, the average value of activation entropy ΔS^≠^ under N_2_ is − 399.54 J mol^−1^ K^−1^, and that under CO_2_ is − 338.92 J mol^−1^ K^−1^. The average value of activation entropy ΔS^≠^ is negative, which indicates that the orderliness increases, and the degree of reaction progress is more difficult to spontaneously accelerate, so it needs additional energy injection into the reaction system. Moreover, the activation entropy ΔS^≠^ of oil shale pyrolysis under CO_2_ is larger than that under N_2_, which indicates that the second stage pyrolysis of oil shale is easier under CO_2_.

### DSC verification of thermodynamic decomposition characteristics

**Figure 12:**
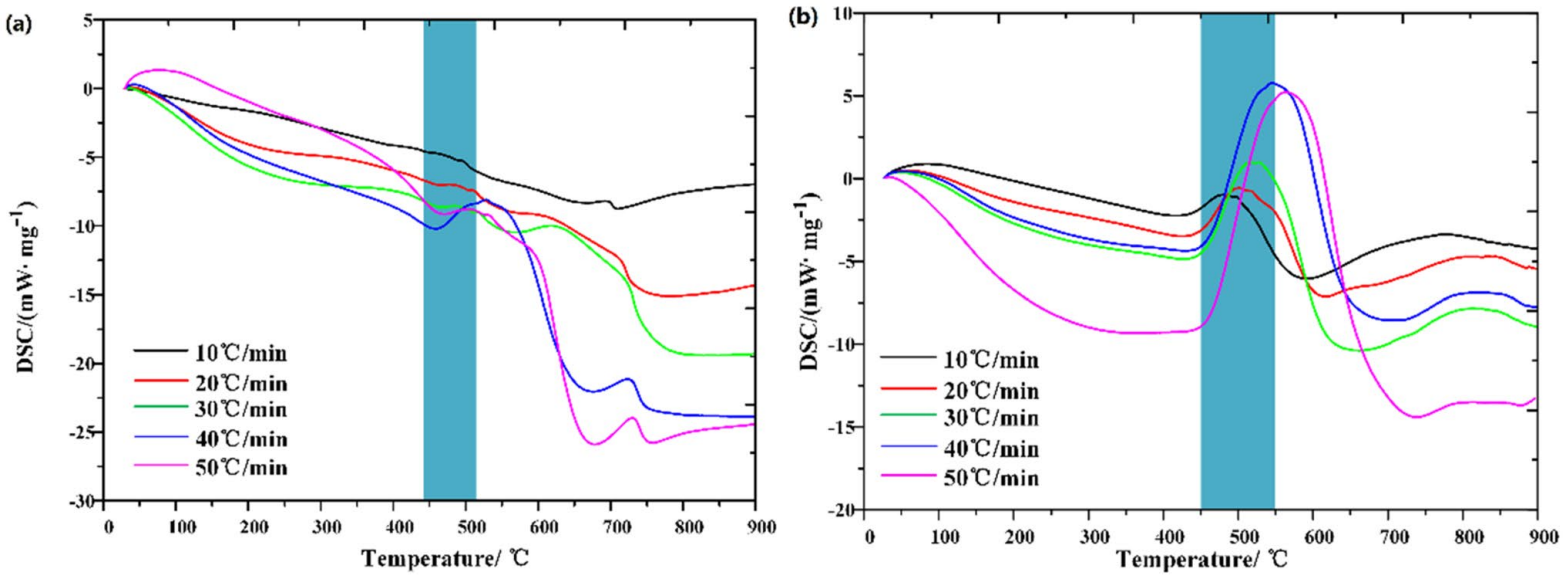
DSC curve of Huadian oil shale (**a**) N_2_ (**b**) CO_2_.

In order to verify the accuracy of thermodynamic calculation, DSC (Differential Scanning Calorimetry) analysis of oil shale pyrolysis was carried out under N_2_ and CO_2_, respectively, as shown in Fig. 12.

**Figure 13:**
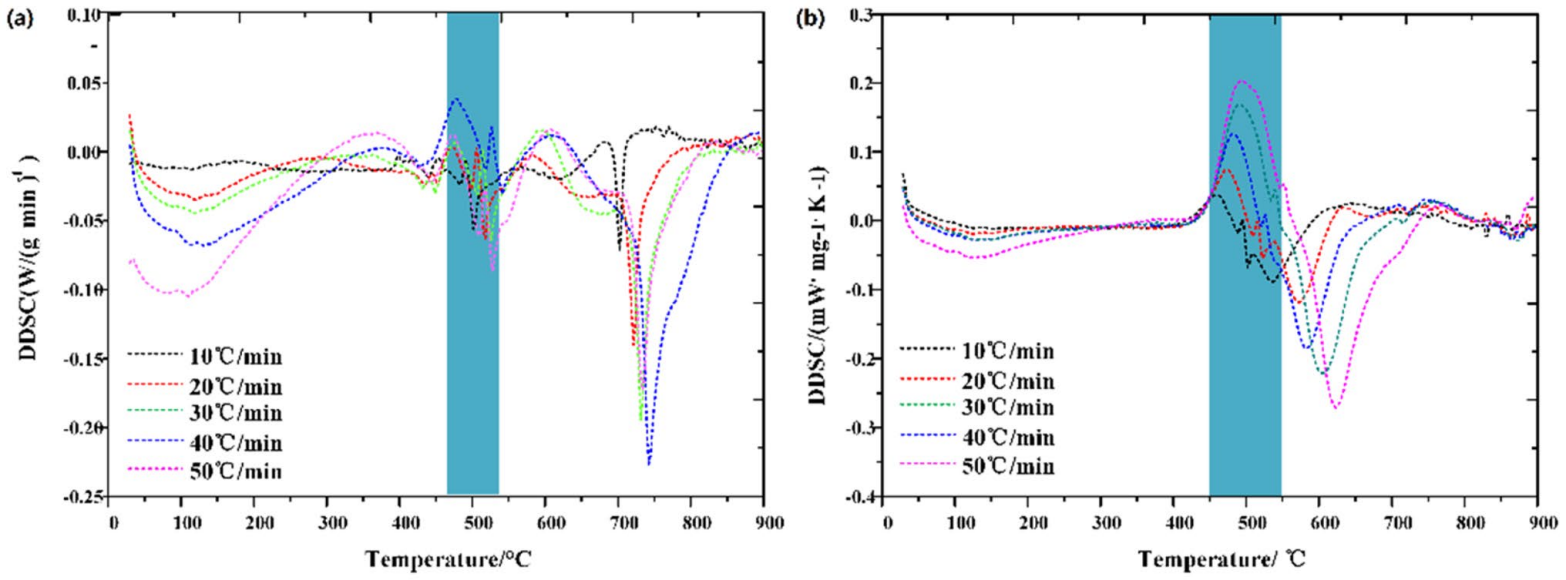
DDSC curve of Huadian oil shale (**a**) N_2_ (**b**) CO_2_.

In the second stage of oil shale pyrolysis under N_2_, the heat release rate slows down. The first-order differential calculation of DSC curve under N_2_ is carried out to obtain the heat release rate curve, as shown in Fig. 13a. In the second stage of oil shale pyrolysis, the endothermic process occurs, because macromolecular hydrocarbons are produced, and the branch chains of macromolecular substances are prone to break chemical bonds at high temperature to produce small molecular substances, which is the secondary decomposition of oil shale. When the displacement efficiency is low, the oil and gas products cannot be eliminated in time, and the degree of secondary decomposition will increase. In this experiment, the degree of secondary decomposition is low, so the endothermic scale is small with the maximum endothermic rate being only 0.043 W/(mg min), and with the increase of heating rate, the endothermic rate also shows an increasing trend.

Under CO_2_, the endothermic process is more obvious, as shown in Fig. 13b. This is because CO_2_ reacts with carbon in oil shale semi coke at high temperatures, i.e.

$$\mathrm{C}+\mathrm{CO}_{2}\mathop \rightarrow\limits^{High \,temperature} \mathrm{CO}$$

**Figure 14:**
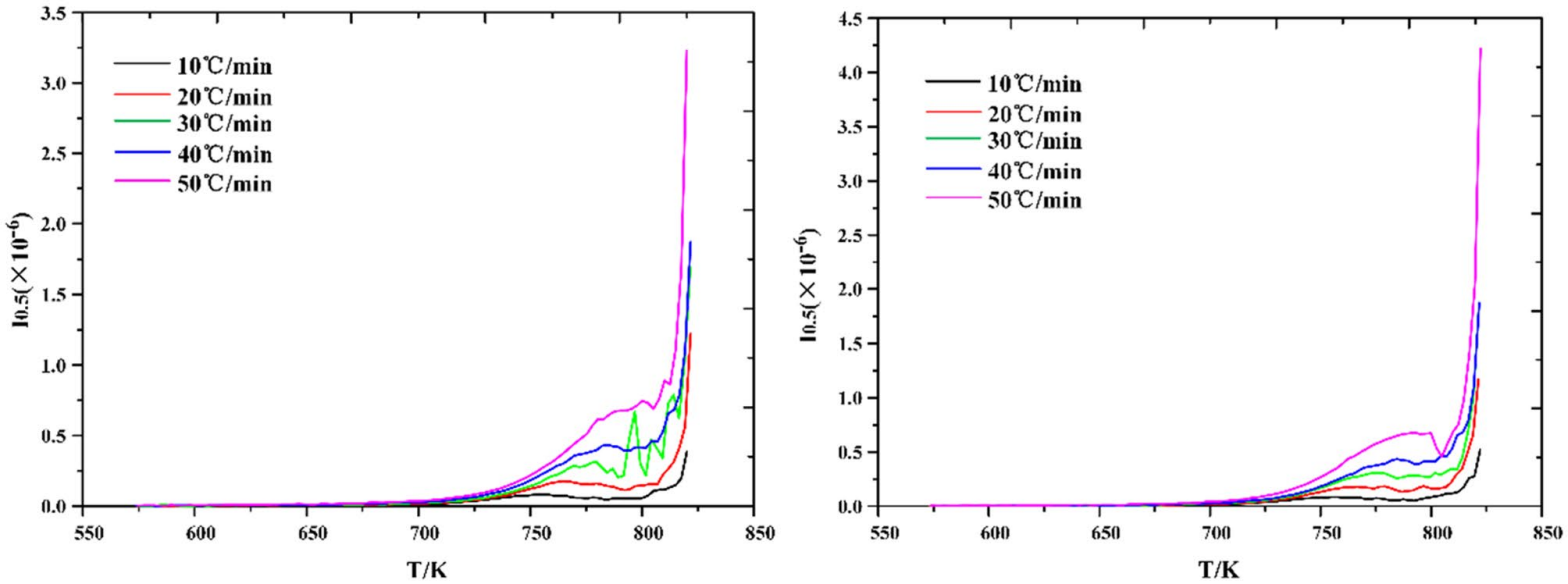
Volatile release characteristic index (**a**) N_2_ (**b**) CO_2_.

This reaction is an endothermic reaction, because the oil shale semi-coke of oil shale exposed to CO_2_ will occur this reaction, so the amount of oil shale reacted is large, so the heat absorption of oil shale pyrolysis is more obvious under CO_2_. In addition, as shown in Fig. 14b, the endothermic rate also tends to increase with the increase of heating rate. Higher endothermic intensity is presented under CO_2_ than that under N_2_ because of the larger quality of oil shale participating in this reaction. This also verifies the accuracy of thermodynamic calculation.

### Volatile release characteristics

In this paper, volatile release index I and reactive index Rα are used to describe the difference of non-isothermal pyrolysis product release characteristics and conversion rate of Huadian oil shale under CO_2_ and N_2_^18, 19^. The conversion of oil shale in the second stage of pyrolysis is calculated at 50%.

21 $${\mathrm{I}}_{1/2}=\frac{{R}_{\alpha }}{{T}_{P}{{T}_{i}\Delta T}_{1/2}}$$

22 $${\Delta T}_{1/2}\to \frac{dm/dt}{{R}_{\alpha }}=\frac{1}{2}$$

23 $${R}_{\alpha }=-\frac{1}{m-{m}_{\infty }}\frac{dm}{dt}=\frac{1}{1-\alpha }\frac{d\alpha }{dt}$$

where, T_i_—ignition point of oil shale obtained by extrapolation method. T_P_—The burnout temperature of oil shale. ∆T_1/2_—The temperature range corresponding to 50% conversion, K; also called peak width. The above announcement reflects the characteristics of volatile matter released by instantaneous organic matter transformation in the process of oil shale pyrolysis to 50%.

As shown in Fig. 14, the volatile release index of oil shale pyrolysis during the second stage is significantly affected by the heating rate. When the heating rate is low, the volatile matter release from oil shale is uniform. With the increase of heating rate, pyrolysis moves to high temperature zone, and the volatile release index increases, indicating that the increase of heating rate will lead to the concentrated release of products. At the same heating rate, the volatile release characteristic index under CO_2_ is higher than that under N_2_. This is because oil shale semi-coke in solid oil shale also reacts with CO_2_ to release carbon monoxide gas, and at high temperatures, oil shale semi-coke exposed to CO_2_ will react. Therefore, in addition to the volatiles produced by oil shale pyrolysis, the reaction between CO_2_ and oil shale semi-coke also releases volatiles, resulting in an increase in the volatile release characteristic index under CO_2_. During the pyrolysis of oil shale, the chemical bond of kerogen group breaks and many free radicals such as hydroxyl, methyl and hydrogen are produced. Under high temperature, these active free radicals recombine. These can accelerate the formation of small molecular compounds with more stable molecular structure. Same finding has been found by Gao Songping^20^, Zhijun Zhou^6^, Prabhat naredi^21^, Reinhard C^22^, Lunbo Duan^23^ also.

### Kinetic compensation effect

The main characteristic of kinetic compensation effect is the activation energy E of chemical reaction, which will be included in the index term and pre exponential factor A of Arrhenius reaction rate constant equation. Activation energy refers to the minimum energy required for the reactant molecules to reach the activation state in chemical reactions. The pre exponential factor is the number of molecules that have an effective collision. The increase of activation energy makes the reaction not easy, but the increase of pre exponential factor can accelerate the reaction rate, so there is a compensation effect between activation energy and pre exponential factor in the process of oil shale pyrolysis.

According to the Arrhenius equation^24, 25^,

$$k=Aexp\left[-E/\left(RT\right)\right]$$

So the kinetic compensation effect is usually expressed by the following equation,

$$\mathrm{ln}A=aE+b$$

where, k—the reaction rate constant; a,b- compensation factors.

**Figure 15:**
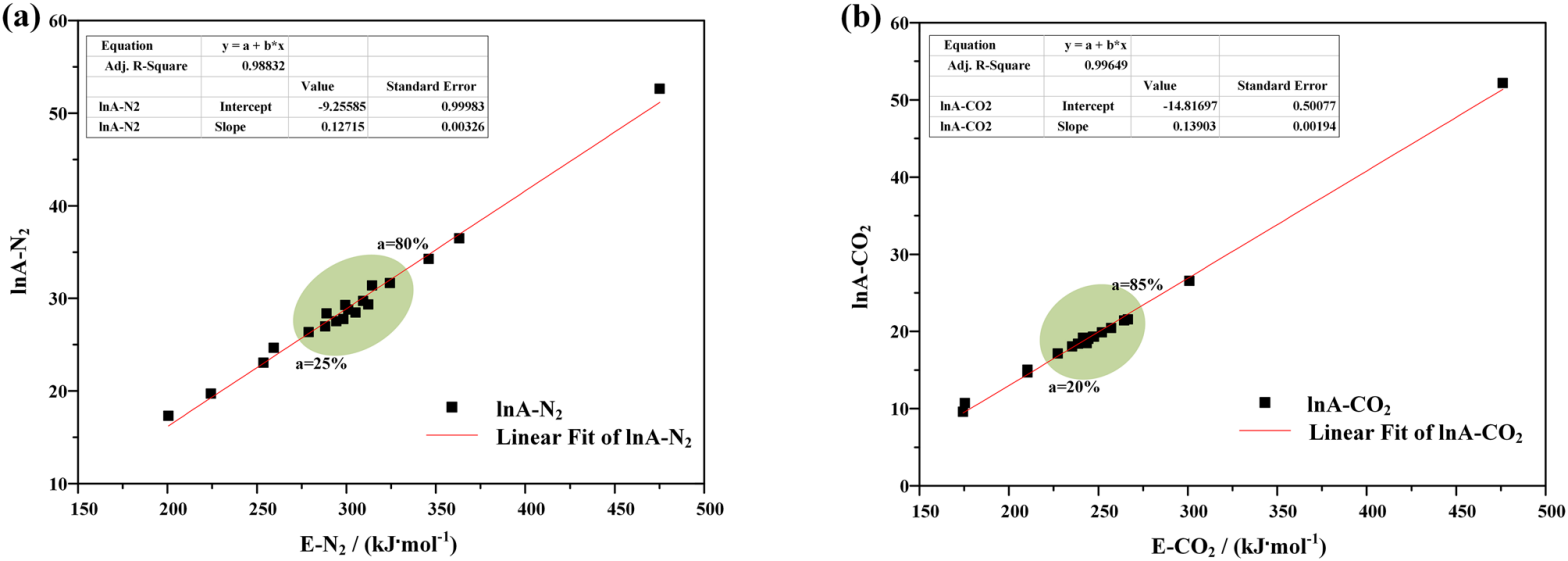
Kinetic compensation effect of oil shale pyrolysis under (**a**) N_2_ and (**b**) CO_2_.

Through the analysis of the kinetic compensation effect, we find that the pre exponential factor increases only when the activation energy increases in nitrogen atmosphere. In order to measure the increase of frequency factor, we choose the range of centralized pyrolysis and calculate the ratio of activation energy to pre exponential factor. The results shows in Fig. 15 that the ratio of activation energy to pre exponential factor is 10.01 ~ 10.72 when the conversion rate is 25% ~ 80% in nitrogen atmosphere, but 12.34 ~ 14.35 when the conversion rate is 20% ~ 85% in carbon dioxide atmosphere. This shows that the compensation effect is stronger in nitrogen atmosphere, but the concentration of volatile matter release index and oil shale pyrolysis is lower. Why? This is because the specific heat capacity of nitrogen is larger than that of carbon dioxide. This leads to a greater heat transfer resistance in the process of oil shale pyrolysis. At the same purging flow rate, the flowing nitrogen takes more heat than carbon dioxide, so the heat transfer resistance is greater. Therefore, the dispersion of oil shale pyrolysis in nitrogen atmosphere is higher, the release index of volatile matter is lower, and the activation energy is higher.

## Conclusion

1. The KAS and FWO methods were used to calculate the activation energy of the second stage of Huadian oil shale pyrolysis. The results show that the average activation energy under N_2_ is about 301.96 kJ mol^−1^, and that under CO_2_ is about 250.28 kJ mol^−1^. From the view of kinetics, the second stage pyrolysis of oil shale is easier under CO_2_.
2. The average activation entropy ΔS^≠^ under CO_2_ is − 338.92 J mol^−1^ K^−1^, which is greater than that − 399.54 J mol^−1^ K^−1^ under N_2_. The average activation enthalpy ΔH^≠^ under N_2_ is about 28.98 kJ mol^−1^, which is less than 76.35 kJ mol^−1^ under CO_2_. From the viewpoint of thermodynamic, the second stage pyrolysis of oil shale is easier under CO_2_.
3. The specific heat capacity of nitrogen is larger than that of carbon dioxide. This leads to a greater heat transfer resistance in the process of oil shale pyrolysis. At the same purging flow rate, the flowing nitrogen takes more heat than carbon dioxide, so the heat transfer resistance is greater. Therefore, the dispersion of oil shale pyrolysis in nitrogen atmosphere is higher, the release index of volatile matter is lower, and the activation energy is higher.
